# Hemobilia in a patient with arteriobiliary fistula after liver contusion

**DOI:** 10.1590/0100-3984.2017.0111

**Published:** 2018

**Authors:** Karen Cristine Pereira Ribeiro, João Paulo de Oliveira Guimarães, Leonardo Branco Aidar, Thiago Adriano da Silva Guimarães, Júlio César Santos da Silva

**Affiliations:** 1 Hospital Regional Antônio Dias (HRAD) - Fundação Hospitalar de Estado de Minas Gerais (FHEMIG), Patos de Minas, MG, Brazil.; 2 Hospital das Clínicas da Faculdade de Medicina da Universidade de São Paulo (HC-FMUSP), São Paulo, SP, Brazil.

**Figure 1:**
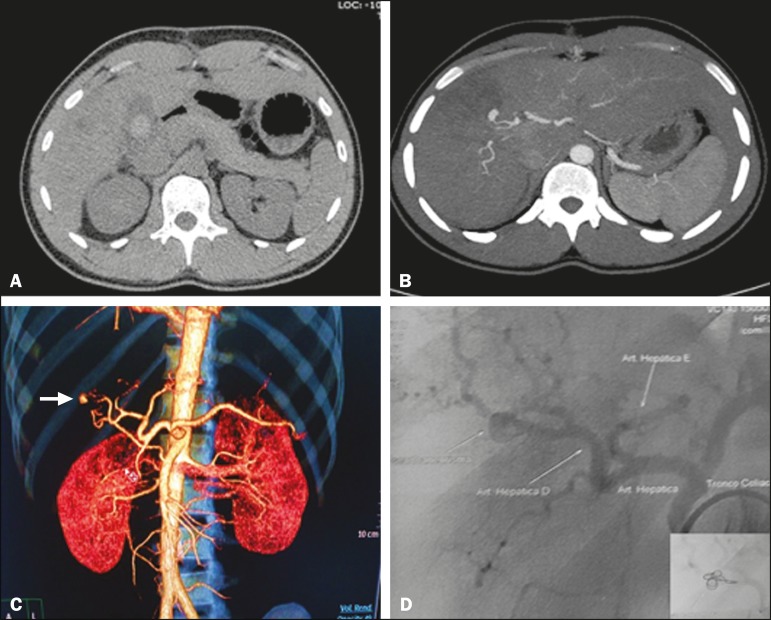
**A:** Noncontrast axial CT showing spontaneously hyperdense (possibly hematic) material in the interior of the gallbladder. **B:** Axial CT after intravenous contrast administration, with MIP, showing arterial lesion due to local liver contusion, with a probable communication between the artery and the biliary duct. **C:** Volumetric reconstruction (arterial phase) showing a pseudoaneurysm/contrast extravasation in a branch of the right hepatic artery (arrow). **D:** Arteriography with embolization and total exclusion of the pseudoaneurysm and of the arterial rupture, the final result being angiographic success.

Dear Editor,

We report the case of a 25-year-old male patient with a history of blunt abdominal trauma (from a motorcycle accident), who presented with abdominal pain. Full abdominal computed tomography (CT) with intravenous contrast administration revealed that the patient had grade 2 liver contusion in the right lobe. Because the patient was hemodynamically stable, we opted for conservative treatment. However, he evolved to hemodynamic instability. An exploratory laparotomy revealed a mosaic pattern of liver injury, which was treated with hepatorrhaphy. On the eighth day after surgery, the patient was in a stable, lucid state and was discharged. However, he returned 17 days later with abdominal pain after a large meal, together with voluminous hematemesis and hypovolemic shock. We then performed abdominal CT angiography (Figure 1), which revealed a pseudoaneurysm in the right hepatic artery, in close proximity to the liver contusion. There was also spontaneously hyperdense content within the gallbladder, suggesting arteriobiliary fistula. Upper gastrointestinal endoscopy showed blood clots and active bleeding in the papilla of Vater, and arteriography (performed at a different facility) confirmed the existence of pseudoaneurysm in the right hepatic artery in the sub-branch of liver segment V, with contrast extravasation suggestive of rupture. Therefore, embolization was carried out.

Hemobilia is an uncommon condition and is one of the differential diagnoses of upper gastrointestinal hemorrhage^(^^1^^)^. There are many causes of hemobilia, such as iatrogenic and accidental traumas, as well as gallstones, inflammation, vascular malformations, and tumors^(^^2^^)^. The clinical manifestations of hemobilia are determined by the quantity and velocity of the hemorrhage within the biliary tract. Its symptoms are jaundice, right hypochondrium pain, and gastrointestinal hemorrhage (ranging from chronic bleeding, resulting in anemia, to massive bleeding with hypotension), and it can develop several months after a trauma^(^^4^^,^^5^^)^.

The improvement of radiological techniques has been fundamental in the diagnosis and treatment of hemobilia, especially in cases of traumatic pseudoaneurysms^(^^3^^)^. In patients with upper gastrointestinal hemorrhage, upper gastrointestinal endoscopy is the examination of choice, because it can identify blood clots in the ampulla of Vater and rule out other causes of bleeding. Ultrasound is a rapid, noninvasive method that is useful and effective in the detection of hemobilia, potentially revealing blood clots or echogenic intraluminal material in the biliary tree or gallbladder. However, contrast-enhanced CT (in the arterial phase) can detect pseudoaneurysms, obstruction of the common biliary duct, and intrahepatic cavities that may require surgical debridement^(^^3^^)^.

For cases of severe gastrointestinal bleeding in which there is a risk of death, the diagnostic procedure of choice is hepatic angiography, because it allows selective embolization of the appropriate vascular branches, preserving maximum liver parenchyma function. Transcatheter arterial embolization is used as an isolated form of treatment or as a way to keep the patient hemodynamically stable for definitive surgery, minimizing morbidity and mortality^(^^6^^,^^7^^)^.

Currently, embolization of the hepatic artery is the gold standard treatment, due to its 80-100% success rate in controlling hemorrhage and its low rates of morbidity and mortality^(^^2^^,^^8^^)^. However, there are reports of fatal hepatic necrosis and the formation of intrahepatic abscess following embolization^(^^8^^)^. Technical failures may occur in cases of anomalous origin of the hepatic artery, previous surgery, vascular tortuosity, or previous ligation of the proximal vessel. In the case presented here, the patient underwent selective transcatheter embolization and remained in outpatient follow-up, with no abdominal pain and with resolution of the hemobilia.
